# Platinum‐based combination chemotherapy triggers cancer cell death through induction of BNIP3 and ROS, but not autophagy

**DOI:** 10.1111/jcmm.14898

**Published:** 2019-12-19

**Authors:** Ling‐Yen Chung, Shye‐Jye Tang, Yi‐Ching Wu, Kai‐Chi Yang, Hui‐Ju Huang, Guang‐Huan Sun, Kuang‐Hui Sun

**Affiliations:** ^1^ Department of Biotechnology and Laboratory Science in Medicine National Yang‐Ming University, and Department of Education and Research Taipei City Hospital Taipei Taiwan; ^2^ Institute of Marine Biotechnology National Taiwan Ocean University Keelung Taiwan; ^3^ Division of Urology Department of Surgery Tri‐Service General Hospital and National Defense Medical Center Taipei Taiwan

**Keywords:** autophagy, BNIP3, combination chemotherapy, reactive oxygen species

## Abstract

These days, cancer can still not be effectively cured because cancer cells readily develop resistance to anticancer drugs. Therefore, an effective combination of drugs with different mechanisms to prevent drug resistance has become a very important issue. Furthermore, the BH3‐only protein BNIP3 is involved in both apoptotic and autophagic cell death. In this study, lung cancer cells were treated with a chemotherapy drug alone or in combination to identify the role of BNIP3 and autophagy in combination chemotherapy for treating cancer. Our data revealed that various combinational treatments of two drugs could increase cancer cell death and cisplatin in combination with rapamycin or LBH589, which triggered the cell cycle arrest at the S phase. Cells with autophagosome and pEGFP‐LC3 puncta increased when treated with drugs. To confirm the role of autophagy, cancer cells were pre‐treated with the autophagy inhibitor 3‐methyladenine (3‐MA). 3‐MA sensitized cancer cells to chemotherapy drug treatments. These results suggest that autophagy may be responsible for cell survival in combination chemotherapy for lung cancer. Moreover, BNIP3 was induced and localized in mitochondria when cells were treated with drugs. The transfection of a dominant negative transmembrane deletion construct of BNIP3 (BNIP3ΔTM) and treatment of a reactive oxygen species (ROS) inhibitor suppressed chemo drug‐induced cell death. These results indicate that BNIP3 and ROS may be involved in combination chemo drug‐induced cell death. However, chemo drug‐induced autophagy may protect cancer cells from drug cytotoxicity. As a result, inhibiting autophagy may improve the effects of combination chemotherapy when treating lung cancer.

## 1 INTRODUCTION

Lung cancer is the leading cause of cancer‐associated deaths worldwide. Two major clinical types of lung cancer have been well identified, which are small‐cell lung cancer (SCLC) and non‐small‐cell lung cancer (NSCLC). Approximately 85% of lung cancer is histologically classified as NSCLC. The 5‐year survival rate of most patients with metastatic NSCLC remains low.^1^ In general, two chemotherapy drugs combined are standard for treating NSCLC. Elderly patients or those in poor health may not tolerate combination chemotherapy and may thus be given single‐drug chemotherapy. Combination chemotherapy frequently contains cisplatin or carboplatin plus pemetrexed, gemcitabine or docetaxel. For patients with advanced lung cancers, a targeted therapy or immunotherapy drug such as bevacizumab, erlotinib or pembrolizumab may be added to the treatment.^1^ However, most patients acquire drug resistance at around 6‐18 months, so continued research on new combination therapies and drug resistance mechanisms can improve the outcomes of NSCLC patients.

Cisplatin (CDDP), which belongs to a class of platinum‐containing antineoplastic drugs, is the first‐line chemotherapy regimen for many kinds of cancers. Cisplatin triggers cell apoptosis by binding to DNA and crosslinking the DNA strands. However, its efficacy is limited due to adverse drug effects and the development of drug resistance. Several strategies can overcome cisplatin drug resistance and toxicity, including liposome delivery, inhibition of glutathione and metallothionein species, and combination therapy.^2^ The mammalian target of rapamycin (mTOR) is a serine/threonine protein kinase that controls cell proliferation and survival. AKT1 overexpression and gene amplification have been shown to induce lung cancer cells to become resistant to cisplatin through mTOR signaling pathway.^3^ Rapamycin, also known as sirolimus, is a well‐studied mTOR inhibitor and has potent immunosuppressive and antiproliferative properties. It is a promising therapeutic agent for solid organ transplantations and cancer treatments.^4^ In addition to the direct antiproliferative effect on tumour cells, rapamycin impedes tumour growth by blocking angiogenesis in a mouse metastatic cancer model.^5^ LBH589 (panobinostat), a non‐selective histone deacetylase inhibitor (pan‐HDAC inhibitor), has been shown to induce tumour shrinkage and sustain stable disease status in a phase II trial of pre‐treated lung cancer patients.^6^ LBH589 is the first HDAC inhibitor to be approved by the FDA to treat multiple myeloma patients.^7^

Autophagy is a self‐digesting catabolic process induced under various stress conditions for homoeostatic cellular recycling that has been implicated in the progression of many diseases, including cancer and neurodegeneration.^8^ Autophagy has been found to play a paradoxical role in anticancer treatments. In some pharmacological studies, autophagy induction is required for immunogenic cancer cell death. On the other hand, activated autophagy mediates drug resistance and survival in some cancer cells.^8^, ^9^ BNIP3 (Bcl‐2/adenovirus E1B 19 kD protein‐interacting protein 3) is a pro‐cell death member of the Bcl‐2 family and contains only the BH3 domain. BNIP3 forms stable homodimerization complexes on the outer membrane of mitochondria to induce apoptosis, necrosis and autophagy after cellular stress.^10^ The carboxy terminal tail of BNIP3, but not BH3, is essential for inducing mitochondrial permeability transition and cytochrome c release, while the transmembrane (TM) domain is required for mitochondrial localization.^11^ BNIP3 expression is down‐regulated by promoter hypermethylation or homozygous gene deletion in certain cancers, whereas their expression increased and were correlated with poor prognosis in other cancers.^10^ Furthermore, the lack of BNIP3 expression has been linked to chemoresistance of pancreatic cancer cells to gemcitabine and 5‐fluorouracil.^12^, ^13^ However, the role of BNIP3 regarding cell death in the combination chemotherapy of lung cancer is not yet properly understood. In this study, we used cisplatin and two novel chemo drugs (LBH589 and Rapamycin) with different molecular mechanisms to treat lung cancer cells. We found that BNIP3 and ROS may be involved in combination chemo drug‐induced cell death and inhibition of autophagy to improve the effects of combination chemotherapy in treating lung cancer.

## 2 MATERIALS AND METHODS

### 2.1 Antibodies, plasmids, and reagents

Cisplatin (cis‐diaminodichloroplatinum (II), CDDP), 3‐methyladenine (3‐MA), N‐acetyl‐L‐cysteine (NAC), acridine orange (AO), propidium iodide (PI), LBH589, bafilomycin A1, tetramethylrhodamine methyl ester perchlorate (TMRM), GAPDH antibody and α‐tubulin antibody were purchased from Sigma‐Aldrich. Rapamycin, LC3 antibody and BNIP3 antibody were obtained from Calbiochem, Novus Biologicals and Abcam, respectively. The pEGFP‐LC3 plasmid was kindly provided by Dr Wei‐Pang Huang (Department of Life Science, National Taiwan University).

### 2.2 Cell culture

Human lung adenocarcinoma A549 (ATCC; no. CCL‐185) cells were obtained from the Bioresource Collection and Research Center (BCRC, Hsinchu City, Taiwan). A549 cells were cultured in an RPMI‐1640 medium (Thermo Scientific Hyclone) supplemented with 10% foetal bovine serum (FBS, Gibco BRL, Life Technologies) and 1% penicillin‐streptomycin (P‐S; Gibco BRL, Life Technologies). Cells were incubated at 37°C in a humidified incubator at 5% CO_2_.

### 2.3 Cell viability

Cells (5 × 10^3^ per well in 96 well plates or 2 × 10^5^ cells per 6‐cm dish) were treated with cisplatin (2 μg/mL), LBH589 (100 nmol/L), rapamycin (100 nmol/L) or a combination of two drugs for 48 hours. Cell viabilities were determined by Celltiter‐Glo Luminescent Cell Viability Assay (Promega) according to the manufacturer's instructions. At the time of assay, CellTiter‐Glo reagent was added and mixed for 2 minutes on an orbital shaker. Luminescent signals were obtained using TECAN sunrise ELISA reader (Tecan, Trading AG) after 10 minutes of incubation.

### 2.4 Cell cycle analysis

Cells (2 × 10^5^ cells per 6‐cm dish) were incubated with cisplatin (2 μg/mL), LBH589 (100 nmol/L), rapamycin (100 nmol/L) or a combination of two drugs for 48 hours. The cells were harvested by trypsinization, washed in PBS and resuspended in 70% ethanol at −20°C. After 30 minutes of incubation, cells were washed twice with PBS. Decanting of all the supernatant was followed by adding 800 μL PBS, 100 μL propidium iodide (400 μg/mL) and 100 μL RNase A (1 mg/mL) to the sample. The cells were incubated for 30 minutes at 37°C in the dark and then analysed using FACS Calibur (BD Biosciences).

### 2.5 Reverse transcription and quantitative polymerase chain reaction (RT‐qPCR)

Total RNA extraction from A549 cells was performed with TRIzol reagent (Ambion, Life Technologies) according to the recommendations of the manufacturer. Then, 5 μg of total RNA was reverse transcribed with the SuperScript III First‐Strand Synthesis System (Invitrogen) to synthesize cDNA samples. Real‐time PCR was performed on an ABI StepOnePlus Real‐Time PCR System (Applied Biosystems) using SYBR Green Master Mix (Applied Biosystems). The primer sequences of *BNIP3*, *BECN1*, *ATG5*, *ATG7* and *GAPDH* were as follows: *BNIP3*: 5′‐TCAAGTCGGCCGGAAAATAT‐3′ (sense), 5′‐GCGCTTCGGGTGTTTAAAGA‐3′ (antisense); *BECN1*: 5′‐TCCTGGACCGTGTCACCAT‐3′ (sense), 5′‐CGCCTGGGCTGTGGTAAGTA‐3′ (antisense); *ATG5*: 5′‐GGAATTGAGCCAATGTTGGAA‐3′ (sense), 5′‐CCGGGTAGCTCAGATGTTCAC‐3′ (antisense); *ATG7*: 5′‐AGCAGCCCACAGATGGAGTAG‐3′ (sense), 5′‐ACGGTCACGGAAGCAAACA‐3′ (antisense); *GAPDH*: 5′‐CAACTACATGGTTTACATGTTC‐3′ (sense), 5′‐GCCAGTGGACTCCACGAC‐3′ (antisense). All values were normalized against the GAPDH, and each sample was assayed in triplicate.

### 2.6 Western blotting

Cells were treated with the drugs cisplatin, LBH589 and rapamycin, either alone or in combination. Cell lysates were extracted into RIPA Lysis buffer (Millipore). Protein concentrations were measured, and equal amounts of total protein were resolved using a 10% sodium dodecyl sulphate‐ polyacrylamide gel electrophoresis (SDS‐PAGE) and then transferred to nitrocellulose membrane (Millipore). The membrane was blocked in 5% skim milk for 1 hour and incubated with the indicated antibodies and horseradish peroxidase‐conjugated secondary antibodies. The immunocomplexes were detected using the chemiluminescence HRP substrate (ECL, Millipore Corporation).

### 2.7 Construction of pEGFP‐BNIP3 and ΔTM‐BNIP3 plasmids

Cloning of BNIP3 and deletion mutant was carried out as described in a previous study.^11^ Briefly, full‐length DNA encoding BNIP3 was amplified by RT‐PCR from human embryonic kidney (HEK) 293 cells and cloned into pEGFP (Clontech Laboratories, Inc). Using the splice overlap extension method,^11^ the transmembrane deletion mutant (BnipΔ164‐183 and ΔTM‐BNIP3) was cloned into pcDNA3 (Invitrogen). Sequences of BNIP3 and the deletion mutant were confirmed by Mission Biotech Co.

### 2.8 Transient transfection

A549 cells were seeded 18 hours prior to transfection with plasmids using jetPEI™ (Polyplus Transfection) transfection reagent according to the manufacturer's instruction. In short, the plasmid and jetPEI™ mixture was added to cells and incubated for 24 hours. Afterwards, the medium was replaced with a fresh, complete medium. For pEGFP‐LC3 transfection, cells were observed under fluorescence microscopy (Leica DM 6000B).

### 2.9 TMRM mitochondrial staining

A549 cells (2 × 10^5^ cells per 6‐cm dish) were transfected with pEGFP‐BNIP3 plasmid. Sixteen hours later, cells were treated with anticancer drugs for 24 hours. Cells were then incubated with 2 nmol/L tetramethylrhodamine methyl ester perchlorate (TMRM) to stain the mitochondria. After 30 minutes, cells were washed three times with fresh PBS and then analysed using fluorescence microscopy (Leica DM 6000B).

### 2.10 Acridine orange (AO) staining

A549 cells (2 × 10^5^ cells per 6‐cm dish) were transfected with pcDNA empty vector or ΔTM‐BNIP3 plasmid. After 16 hours of transfection, cells were incubated with cisplatin (2 μg/mL) alone, LBH589 (100 nmol/L) or rapamycin (100 nmol/L), or with a combination of two drugs for 48 hours. Cells were then stained with acridine orange (AO) solution at a final concentration of 1 μg/mL for 20 minutes at room temperature in the dark and were washed three times with PBS. Images of cells with acidic vesicular organelle (AVO) were visualized with a fluorescence microscope and then photographed. The acidic autophagic vacuoles of stained cells fluoresced bright red, while the cytoplasm and nucleus fluoresced bright green. Furthermore, to quantify the number of cells with AVO, cells were stained with AO (1 μg/mL) at 37°C for 20 minutes. After washing three times with PBS, cells were trypsinized with trypsin‐EDTA, suspended in phenol red‐free RPMI medium and then analysed immediately using flow cytometry.

### 2.11 Statistical analysis

Data are shown as mean ± standard deviation of three independent experiments, and statistical significance was assessed by Student's *t* test. Differences with a *P*‐value of < .05 were considered to be statistically significant.

## 3 RESULTS

### 3.1 Combination of two anticancer drugs with different mechanisms induced cancer cell death more severely

**Figure 1:**
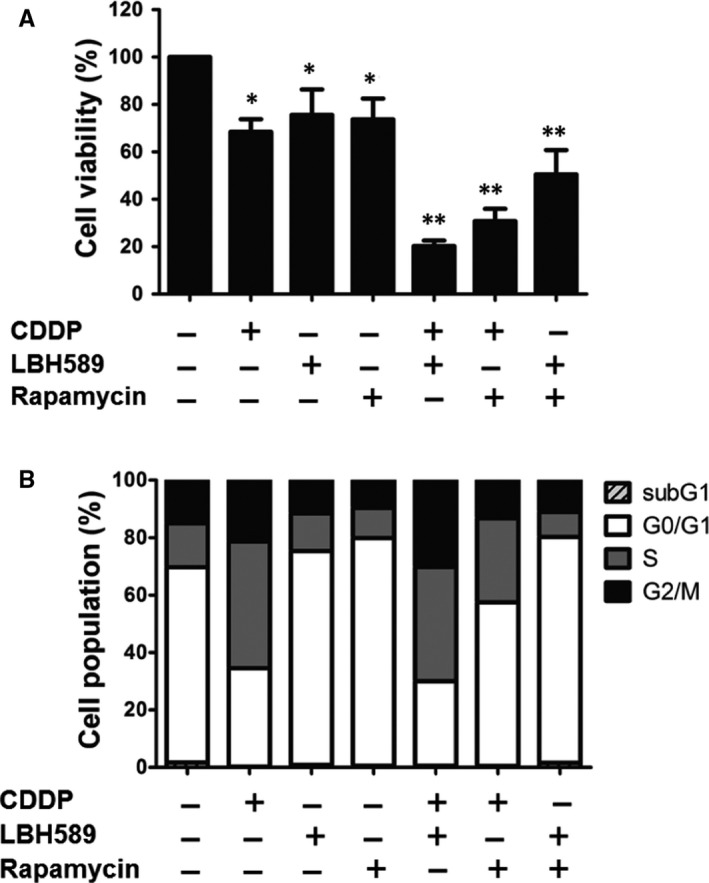
Combination of two anticancer drugs with different mechanisms induced cancer cell death more severely. A549 cells (5 × 10^3^) were treated with cisplatin (2 μg/mL), LBH589 (100 nmol/L), rapamycin (100 nmol/L), or a combination of two drugs for 48 h. A, Cell viability was analysed using a Celltiter‐Glo luminescent cell viability assay. B, Cells were stained with propidium iodide (10 μg/mL) for 30 min for cell cycle analysis by flow cytometry. CDDP, cisplatin. Results are representative of at least three independent experiments. *P < .05 and **P < .01 compared with untreated cells

First, to clarify whether a combination of anticancer drugs with different mechanisms could induce higher cell death of lung cancer cells, we used a DNA damaging agent (cisplatin (CDDP), HDAC inhibitor (LBH589) and mTOR inhibitor (rapamycin). A549 cells were treated with these drugs alone or in combination, and then their cell viability was measured. As shown in Figure 1A and Table 1, cisplatin, LBH589 and rapamycin treatment alone all decreased cell viability. Combinations of any of the two drugs induced cell death at a higher rate than that of the same doses used alone. Notably, the combination of cisplatin and LBH589 exhibited the greatest synergistic effect. We used flow cytometry to further assess the effects of chemotherapy drugs on the cell cycle. Interestingly, in addition to increasing cells in the G2/M phase, a large amount of cells in the S phase was induced upon cisplatin treatment. However, LBH589 or rapamycin treatment alone slightly increased G0/G1 phase cells compared with control. Furthermore, cisplatin/LBH589 co‐treatment increased the arrested cell cycle of the S and G2/M phases when compared with the control group (Figure 1B). Cisplatin/rapamycin co‐treatment increased S phase cells, but LBH589/rapamycin co‐treatment showed a similar cell cycle pattern as individual treatment.

**Table 1:** Synergistic effects in two‐drug combination assay

Drug combination	CI
CDDP and LBH589	3.96E‐03 ± 5.96E‐04
CDDP and Rapamycin	6.09E‐03 ± 2.60E‐04
LBH589 and Rapamycin	9.04E‐03 ± 7.31E‐04

Note

CI (combination index) value of <1, 1, and >1 indicates synergism, addictive effect, and antagonism, respectively.

### 3.2 The induction and mitochondrial localization of BNIP3 were identified upon anticancer drug treatment

**Figure 2:**
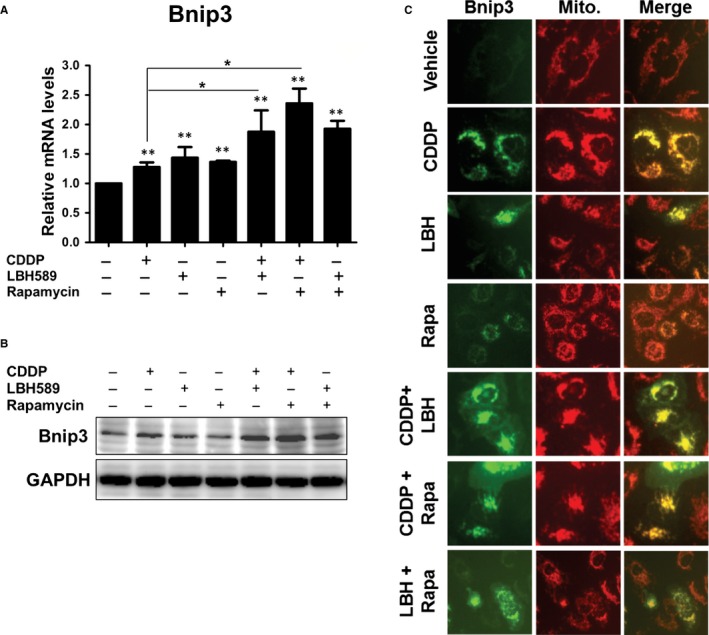
Increased levels and mitochondria localization of BNIP3 were identified upon treatment with anticancer drugs. A, B, A549 cells (2 × 10^5^) were treated with cisplatin (2 μg/mL), LBH589 (100 nmol/L), or rapamycin (100 nmol/L), or a combination of two drugs for 24 h (A) or 48 h (B). The mRNA or protein levels of BNIP3 were detected by qRT‐PCR (A) or Western blot (B), respectively. C, A549 cells were transfected with pEGFP‐BNIP3 plasmid. Sixteen hours later, cells were treated with anticancer drugs for 24 h. Fluorescent dye‐labelled mitochondria were detected using fluorescence microscopy upon treatment of 2 nmol/L of tetramethylrhodamine methyl ester perchlorate (TMRM) for 30 min. Results are representative of at least three independent experiments. **P < .01 compared with untreated cells

AsBNIP3 has been reported as an important molecule in mitochondrial autophagy and cell death,^10^ we aimed to identify whether BNIP3 is involved in anticancer drug‐induced cell death. We found increased levels of BNIP3 in cisplatin, LBH589 and rapamycin treatment in A549 cells. The combinations of any two drugs showed a higher increase in expression of BNIP3, both in mRNA and protein levels, when compared with individual chemotherapy drug treatment (Figure 2A,B). Previous studies have demonstrated that BNIP3 localizes to the mitochondrial membrane. To examine the localization of BNIP3 in anticancer drug‐treated cells, cells were transient transfected with pEGFP‐BNIP3 plasmid, treated with the drugs and then stained with TMRM, a red‐orange fluorescent dye accumulated in mitochondria. The fluorescence analysis clearly showed that the expression of BNIP3 was dramatically up‐regulated in the chemo drug combination regimen, especially in cisplatin‐treated groups, which was co‐localized with mitochondria. These findings indicated that anticancer drug‐induced BNIP3 localized in mitochondria (Figure 2C).

### 3.3 Anticancer drugs induced mitochondrial membrane insertion of BNIP3, resulting in the generation of ROS and cell death

**Figure 3:**
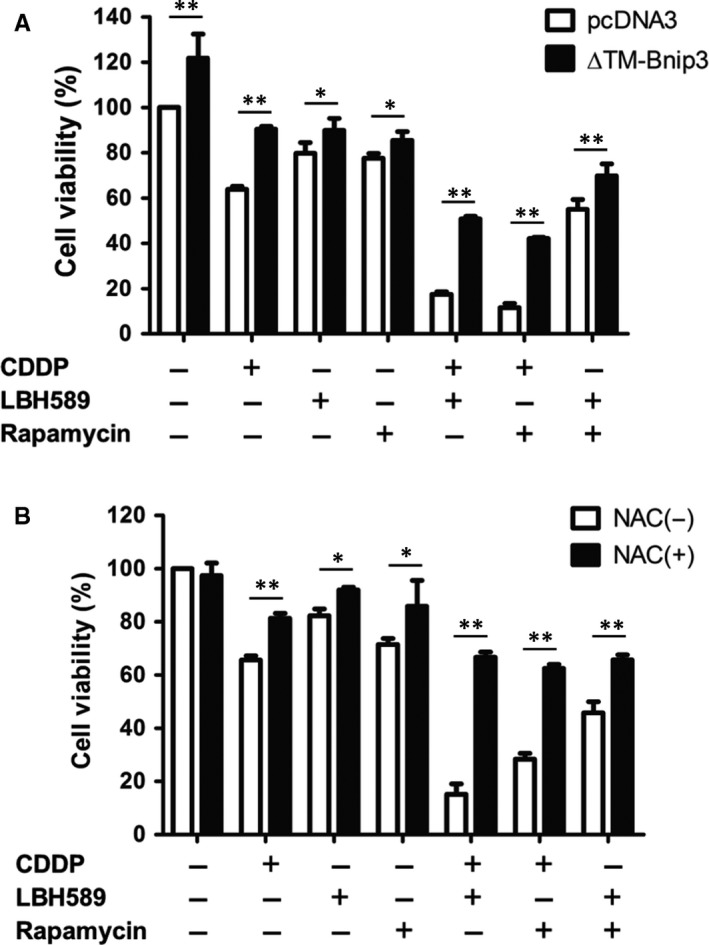
Anticancer drugs induced mitochondrial membrane insertion of BNIP3, resulting in the generation of ROS and cell death. A, A549 cells (2 × 10^5^) were transfected with pcDNA empty vector or ΔTM‐BNIP3 plasmid. Sixteen hours later, cells were treated with cisplatin (2 μg/mL), LBH589 (100 nmol/L), or rapamycin (100 nmol/L), or a combination of two drugs for 48 h. Cell viability was analysed using a Celltiter‐Glo luminescent cell viability assay. B, A549 cells were treated with cisplatin (2 μg/mL), LBH589 (100 nmol/L), or rapamycin (100 nmol/L), or a combination of two drugs for 48 h. Cells were treated with or without the ROS inhibitor NAC (5 mmol/L) 24 h prior to analysis. Cell viability was analysed using a Celltiter‐Glo luminescent cell viability assay. Results are representative of at least three independent experiments. *P < .05 and **P < .01

To further demonstrate whether the synergistic effects of anticancer drug combination were to the result of BNIP3, we constructed a plasmid with mutation in the transmembrane domain of the BNIP3 protein (ΔTM‐BNIP3), which disabled BNIP3 dimerization and insertion into the mitochondria membrane.^10^ After cisplatin, LBH589, and rapamycin treatment alone, cell viability was up‐regulated in ΔTM‐BNIP3‐transfected cells compared with pcDNA3 control cells. Furthermore, cell viability increased much more in ΔTM‐BNIP3‐transfected cells when combining two chemotherapy drugs (Figure 3A). These results indicated that the induction of BNIP3 insertion into the mitochondria membrane sensitized cells to chemo drug‐induced cell death. Previous studies have reported that BNIP3 stimulates ROS (reactive oxygen species) production in cells.^10^ Therefore, to examine whether ROS affect chemo drug‐induced cell death, cells were treated with N‐acetylcysteine (NAC), a widely used ROS scavenger, and assayed cell viability. As shown in Figure 3B, inhibition of ROS significantly decreased the cell death induced by anticancer drug treatment, either alone or in combination. The synergistic effect of chemo drug combinational treatment on cell death was abolished when cells were treated with NAC. These revealed that the induction of BNIP3 and ROS was involved in anticancer drug‐induced cell death.

### 3.4 Chemotherapy drugs induced autophagy in lung cancer cells

**Figure 4:**
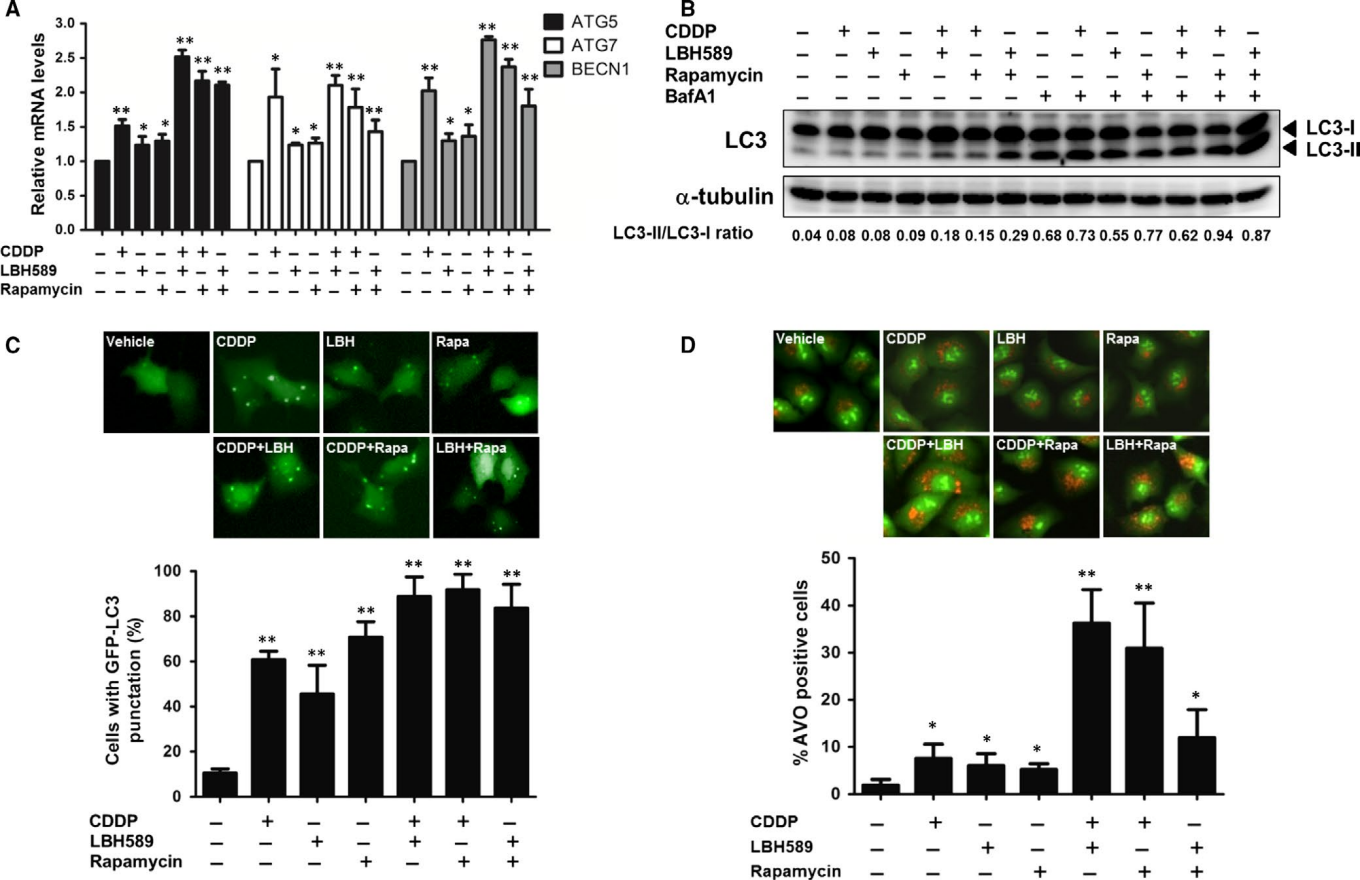
Anticancer drugs induced autophagy in lung cancer cells. A, A549 cells (2 × 10^5^) were treated with the chemotherapeutic drugs cisplatin (2 μg/mL), LBH589 (100 nmol/L), or rapamycin (100 nmol/L), or a combination of two drugs for 24 h. The mRNA levels of *ATG5*, *ATG7* and *BECN1* were assayed by RT‐qPCR. B, chemotherapeutic drug‐induced cells were pre‐treated with or without Bafilomycin A1 (BafA1, 20 nmol/L) for 1 h followed by Western blot using anti‐LC3 antibody. C, A549 cells were treated with chemotherapeutic drugs for 48 h after transfection with pEGFP‐LC3 plasmid. LC3 punctate‐positive cells were observed by fluorescence microscopy and quantitation of the percentage of cells with punctate GFP‐LC3 fluorescence per total GFP‐LC3‐positive cells. Data represent mean ± SD calculated from three experiments of 100 transfected cells each. D, chemotherapeutic drug‐treated cells were stained with AO (1 μg/mL) for 20 min. Autophagic cells were analysed using flow cytometry. Results are representative of at least three independent experiments. *P < .05 and **P < .01 compared with untreated cells

To illustrate whether anticancer drugs can induce autophagy, we analysed the autophagy‐related genes by RT‐qPCR. As expected, treatment of cells with cisplatin, LBH589 and rapamycin alone up‐regulated the expressions of *ATG5*, *ATG7* and *BECN1*. Combinational treatment clearly enhanced the increase of autophagy‐related gene expression (Figure 4A). During the autophagic process, the LC3‐I protein is cleaved to form LC3‐II in autophagosomes. Therefore, cells were treated with anticancer chemo drugs, and the expression of LC3‐II, a well‐characterized marker of cell autophagy, was analysed using Western blot. We observed an increase of the LC3‐II/LC3‐I ratio in cells treated with a combination of two drugs when compared with treatment with a single drug alone (Figure 4B). The induction of LC3‐II was also enhanced in the presence of bafilomycin A1, a known inhibitor of the late‐phase of autophagy. The formation of punctate spots in GFP‐LC3‐transfected cells is also an established marker for visualizing autophagosomes. As the result shows in Figure 4C, anticancer drugs significantly induced the formation of GFP‐LC3 puncta spots. The percentage of cells with GFP‐LC3 punctation was increased when cells were treated with a combination of two drugs.

Acridine Orange (AO) is commonly used to investigate the level of acidic granule formation within cells undergoing autophagy.^14^ The acidic autophagic vacuoles of stained cells fluoresced bright red, while the cytoplasm and nucleus fluoresced bright green. Therefore, cells were incubated with AO stain 20 minutes prior to performing image and flow cytometry analysis. We observed that chemo drug treatment induced the formation of acidic autophagic vacuoles. The induction was largely enhanced when cells were treated with combinational drugs (Figure 4D). These data indicated that anticancer drugs were effective in inducing the autophagy of lung cancer cells.

### 3.5 Autophagic inhibitor 3‐MA suppressed chemotherapeutic drug‐induced autophagy

**Figure 5:**
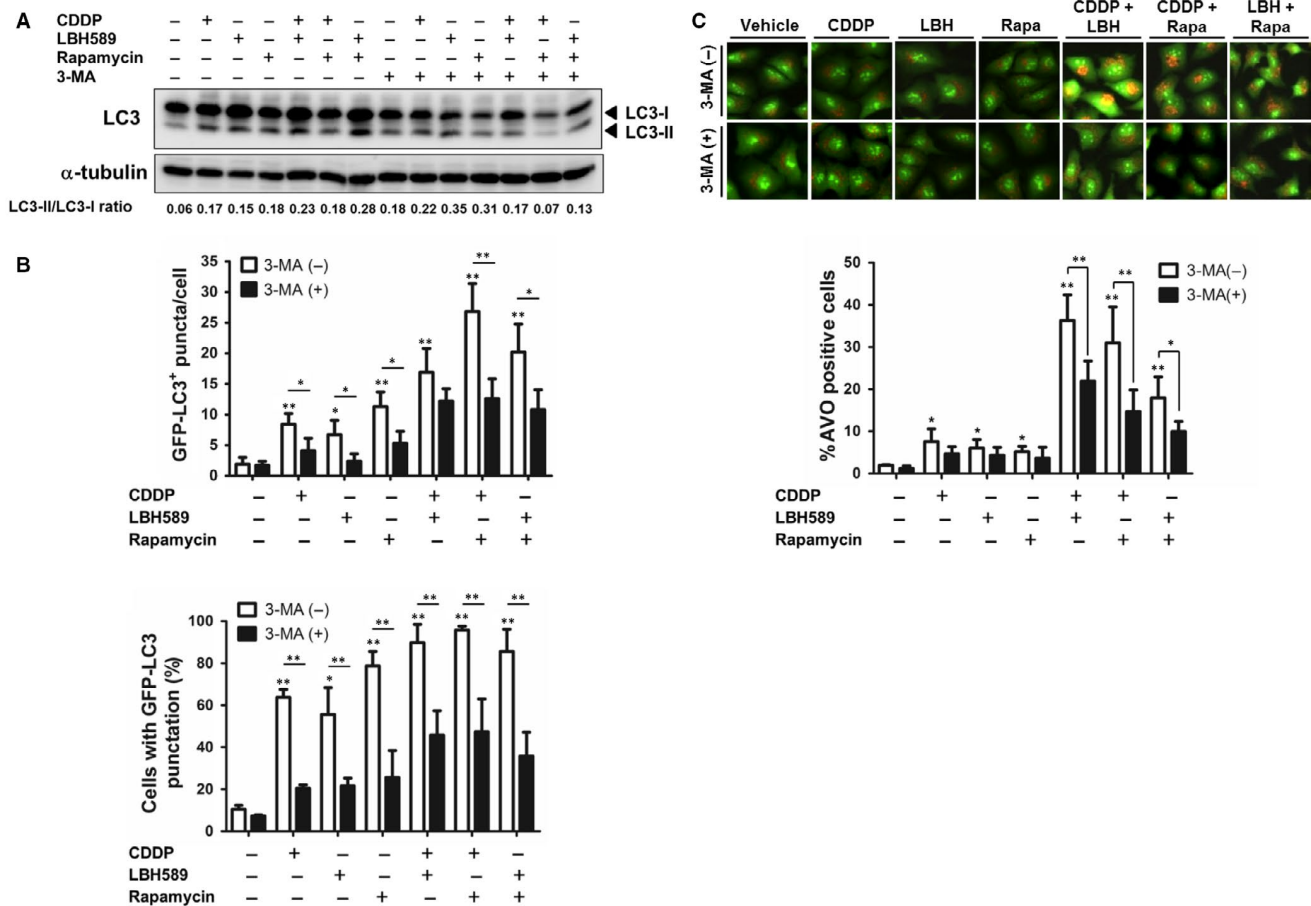
Autophagic inhibitor 3‐MA suppressed chemotherapeutic drug‐induced autophagy. A, A549 cells (2 × 10^5^) were treated with cisplatin (2 μg/mL), LBH589 (100 nmol/L), or rapamycin (100 nmol/L), or a combination of two drugs for 48 h. Cells were treated with or without 3‐MA (3 mmol/L) 1 h prior to analysis. Western blot was conducted using an anti‐LC3 antibody. B, C, pEGFP‐LC3‐transfected A549 cells were treated with chemotherapeutic drugs with or without 3‐MA 1 h prior to analysis. B, Quantitation of the number of GFP‐LC3 puncta per cell was conducted using MetaMorph software. C, LC3 punctate‐positive cells were quantitated by cells with punctate GFP‐LC3 fluorescence per total GFP‐LC3‐positive cells. D, A549 cells were treated with chemotherapeutic drugs with or without 3‐MA 1 h prior to analysis, followed by staining with AO (1 μg/mL). Autophagic cells were analysed using flow cytometry. Results are representative of at least three independent experiments. *P < .05 and **P < .01

To determine whether chemo drug‐induced autophagy can be inhibited by autophagic inhibitor 3‐MA, A549 cells were pre‐treated with 3‐MA and then treated with a single chemo drug alone or in combination and analysed using Western blot. As expected, the LC3‐II/LC3‐I ratio elevations exhibited in combinational treatment groups were abolished when cells pre‐incubated with 3‐MA (Figure 5A). Furthermore, 3‐MA significantly decreased the number of puncta and percentage of cells with punctuation in anticancer drug‐treated cells (Figure 5B). 3‐MA also largely decreased the formation of acidic autophagic vacuoles induced by combinational chemo drug treatment (Figure 5C). These results demonstrated that 3‐MA efficiently suppressed chemotherapeutic drug‐induced autophagy.

### 3.6 Inhibition of autophagy augments chemotherapeutic drug‐induced cell death

**Figure 6:**
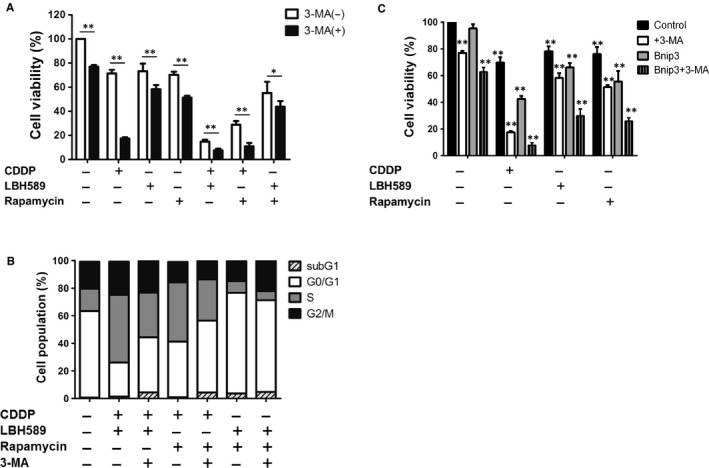
Inhibition of autophagy augments chemotherapeutic drug‐induced cell death. A, B, A549 cells (2 × 10^5^) were treated with cisplatin (2 μg/mL), LBH589 (100 nmol/L), or rapamycin (100 nmol/L), or a combination of two drugs for 48 h. Cells were treated with or without 3‐MA (3 mmol/L) 1 h prior to analysis. A, Cell viability was analysed with a Celltiter‐Glo luminescent cell viability assay. B, Cells were stained with propidium iodide (10 μg/mL) for 30 min. The cell cycle was determined using flow cytometry. C, A549 cells were transfected with pcDNA empty vector or BNIP3 plasmid. Sixteen hours later, A549 cells were treated with chemotherapeutic drugs with or without 3‐MA (3 mmol/L) 1 h prior to analysis. Cell viability was analysed with a Celltiter‐Glo luminescent cell viability assay. Results are representative of at least three independent experiments. *P < .05 and **P < .01

**Figure 7:**
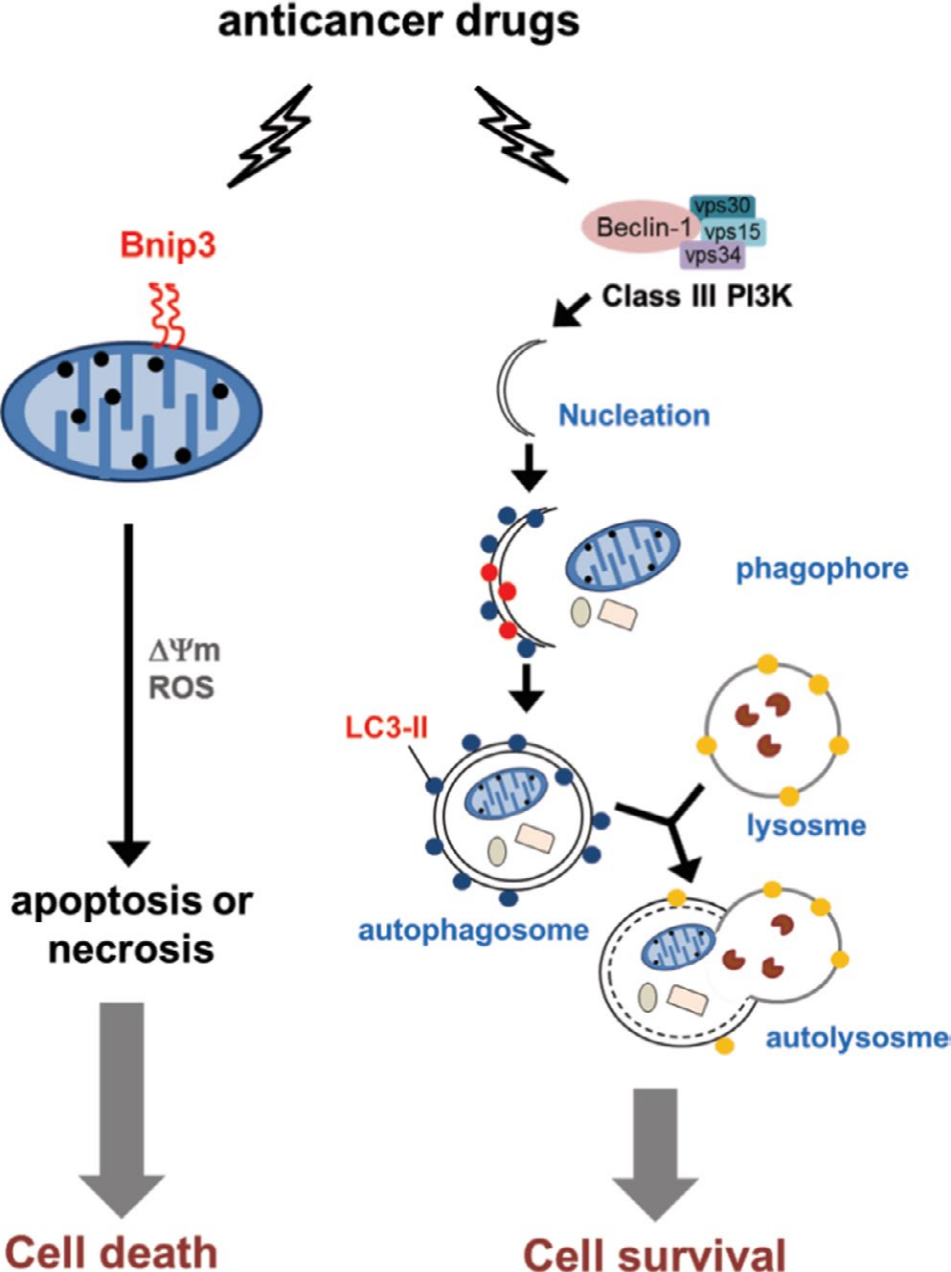
A proposed model for the role of BNIP3 and autophagy in chemotherapeutic drug‐induced cell death. Anticancer drugs augment Binp3 and autophagy in lung cancer cells. Anticancer drugs induction of mitochondrial membrane insertion of BNIP3 may promote ROS and potential membrane loss, resulting in apoptotic or necrotic cell death. However, anticancer drug‐induced autophagy may also be involved in cell survival

To investigate whether chemotherapy drug‐induced autophagy leads to cell survival or cell death, we detected the viability of cells pre‐treated with or without 3‐MA and chemotherapy drugs. We found that cells pre‐treated with 3‐MA significantly promoted cell death in single chemo drug alone or in combination groups (Figure 6A). To further illustrate whether the induction of cell death results from changes in the cell cycle, cells were stained with propidium iodide and analysed with flow cytometry. The results showed that cisplatin in combination with either LBH589 or rapamycin induced cell cycle arrest in the S phase, while the effect was decreased after 3‐MA treatment (Figure 6B). These revealed that chemo drug‐induced autophagy indeed protects A549 cells from death. As our present data showed that autophagy helps lung cancer cells survive chemo drug treatment, we wanted to determine whether inhibition of autophagy can enhance BNIP3‐mediated cell response to the cytotoxic effect of chemo drugs. Cells were transfected with BNIP3 expressing plasmid followed by 3‐MA pre‐treatment and then incubated with anticancer drugs. As shown in Figure 6C, after cisplatin, LBH589 or rapamycin treatment, cells with BNIP3 transfection exhibited a decrease in cell viability when compared with pcDNA control. Furthermore, it showed a synergistic inhibitory effect of BNIP3 overexpression and 3‐MA addition with regard to sensitizing cells to chemotherapy drugs. Therefore, in addition to chemotherapeutic drugs, a combination of BNIP3 activation and autophagy inhibition may be a better strategy for lung cancer therapy. A proposed model for the role of BNIP3 and autophagy in chemotherapeutic drug‐induced cell death is shown in Figure 7. Anticancer drugs augment Binp3 and autophagy in lung cancer cells. Anticancer drugs induction of mitochondrial membrane insertion of BNIP3 may promote ROS and potential membrane loss, resulting in apoptotic or necrotic cell death. However, anticancer drug‐induced autophagy may also be involved in cell survival.

## 4 DISCUSSION

Poor prognosis of NSCLC is mainly due to initial diagnosis occurring at an advanced stage. Despite progress in new therapies, truly effective therapy is still vital for improving the survival of NSCLC patients.^1^ Combination therapy targeting different molecular key pathways is usually selected to enhance therapeutic efficacy and reduce drug resistance of cancer patients in clinics.^15^ Pathways involved in tumour growth that can be targeted include antioxidants, growth factors, angiogenesis, epigenetic modification and signalling pathway. Platinum‐based doublet cytotoxic therapy is the standard therapy for advanced‐stage NSCLC patients. However, only cisplatin combined with pemetrexed showed significant differences in clinical outcome among the multiple cytotoxic treatments for advanced NSCLC patients.^1^, ^16^ In this study, we combined cisplatin with other new pathway inhibitors, mTOR and HDAC inhibitors, to treat NSCLC cancer cells. We found that cisplatin (CDDP) plus HDACi (LBH589) had a greater synergistic effect than cisplatin plus mTOR inhibitor (rapamycin) on cell growth inhibition. Furthermore, LBH589 combined with rapamycin had less effect on cancer cell growth.

Epigenetic regulations of gene expression, including DNA methylation, histone acetylation and methylation, non‐coding RNAs and post‐translational modifications, are crucial drivers for cancer initiation and progression.^17^ Histone deacetylation leads to chromatin decondensation and promotes gene transcription, especially oncogenes and DNA repair genes. Despite approval for T‐cell lymphoma and multiple myeloma, targeting histone deacetylase (HDAC) is a novel treatment for various malignancies and has been explored in phase II/III clinical trials.^18^ HDAC inhibitors have been demonstrated to inhibit tumour growth, induce cell cycle arrest and promote cell apoptosis. Recently, HDAC inhibitors in phase I and II trials have been shown to be well tolerated for advanced NSCLC^19^ and be beneficial for relapsed NSCLC patients,^20^ respectively. LBH589 (panobinostat) has been demonstrated to induce tumour shrinkage and sustain stable disease status in patients with small‐cell lung cancer.^6^ We propose that a combination therapy with HDAC inhibitors will open a new era for chemotherapeutic regimens of NSCLC to enhance efficacy and reduce drug resistance.

mTOR hyperactivation promotes the cell growth and metabolism that contributes to tumour progression in many cancers.^4^ However, mTOR signalling negatively regulates autophagy induction. The PI3K/Akt pathway is the upstream of mTOR signalling and is activated in 50%‐70% of NSCLC patients due to genetic alterations, such as constitutive activation of EGFR and KRAS.^4^ In addition to rapamycin, mTOR inhibitor (everolimus) has demonstrated clinical activity in a phase II study of metastatic NSCLC patients pre‐treated with chemotherapy (CT) or CT and EGFR inhibitors.^21^ Furthermore, mTOR inhibitor (temsirolimus) has also shown benefits for advanced NSCLC patients.^22^ Although rapamycin and its analogues have some adverse effects, clinical trials are still in progress in mTOR inhibitors plus standard therapy for advanced NSCLC.

Autophagy plays a pro‐tumoural or anti‐tumoural role in different stages for cancer progression.^8^ In normal cells, autophagy is activated under metabolic or oxidative stress to suppress cell transformation and tumour initiation. Furthermore, autophagy down‐regulates the epithelial‐mesenchymal transition (EMT)‐promoting transcription factors to inhibit cancer cell motility. However, the quick growth of primary tumours requires autophagy up‐regulation to resist microenvironment low nutrient stress and anoikis.^8^ BNIP3 modulating both pro‐survival and pro‐death effects are dependent on the cell type and microenvironment. C‐terminal TM domain of BNIP3 is essential for homodimerization, proapoptotic function and mitochondrial translocalization. After anchoring into the outer membrane of mitochondria, BNIP3 dimers interact with LC3 through N‐terminal LIR (LC3 interacting region) to induce autophagy.^23^ BNIP3 pro‐death activity depends on the dephosphorylation of the BNIP3 C‐terminus. However, the phosphorylation status of the BNIP3 C‐terminus does not affect the ability of BNIP3 to activate autophagy. Therefore, levels of the BNIP3 C‐terminus phosphorylation control the pro‐survival and pro‐death activities of BNIP3 in response to extracellular stress.^24^ In the present study, we found that the C‐terminal TM domain of BNIP3 was involved in chemo drug‐induced cell death (Figure 3). Nevertheless, the role of BNIP3 C‐terminal phosphorylation in chemo drug‐induced cell death still warrants further investigation.

The role of ROS in cancer development is paradoxical. In the physiological condition, ROS are mainly generated from mitochondria in aerobic cellular metabolism and subsequently degraded by an antioxidant system to prevent cytotoxicity. During the oncogenic transformation of tumour cells, persistent metabolic oxidative stress promotes genomic instability and cancer development.^25^ Although elevated levels of ROS are involved in cancer progression, higher levels of ROS induced by chemotherapy result in cancer cell death.^26^ Therefore, the quantitative monitoring of dynamic ROS levels in tumours during chemotherapy is vital for effective cancer treatment.^26^ In conclusion, our results demonstrated that BNIP3 and ROS are involved in platinum‐based combination chemotherapy, while chemo drug‐induced autophagy may protect cancer cells from cytotoxicity. Therefore, applying autophagy inhibitors may improve the effects of combination chemotherapy in treating lung cancer.

## CONFLICT OF INTEREST

The authors declare that they have no conflicts of interest in relation to this study.

## AUTHOR CONTRIBUTIONS

S‐J Tang, G‐H Sun and K‐H Sun contributed to conception and design of this manuscript; L‐Y Chung, K‐C Yang and H‐J Huang contributed to acquisition of data; Y‐C Wu, S‐J Tang and K‐H Sun contributed to analysis and/or interpretation of data; G‐H Sun and K‐H Sun contributed to drafting and revision of the manuscript.

## Data Availability

Research data are not shared.
